# Altered Neuromuscular Control and Beta-Band Cortical Compensation During Gait in Sarcopenia: An Exploratory Study

**DOI:** 10.3390/bioengineering13060650

**Published:** 2026-05-30

**Authors:** Zengguang Wang, Binbin Wang, Xiaoyan Zhang, Dongyun Gu

**Affiliations:** 1 Shanghai Key Laboratory of Orthopaedic Implants and Department of Orthopaedic Surgery, Shanghai Ninth People’s Hospital, Shanghai Jiao Tong University, Shanghai 200011, China; 2 School of Biomedical Engineering & Med-X Research Institute, Shanghai Jiao Tong University, Shanghai 200030, China; 3 Department of Geriatrics, Shanghai Sixth People’s Hospital Affiliated to Shanghai Jiao Tong University School of Medicine, Shanghai, 200233, China

**Keywords:** sarcopenia, gait dysfunction, beta-band activity, neuromuscular alterations, inefficient compensation

## Abstract

Sarcopenia is an age-related condition characterized by a decline in skeletal muscle mass and function, leading to impaired mobility and an increased risk of adverse health outcomes. However, the neuromuscular mechanisms underlying gait dysfunction in sarcopenia remain incompletely understood. In this study, individuals with sarcopenia and age-matched healthy controls were recruited. Gait parameters were assessed using a motion capture system and quantified through spatiotemporal analysis, muscle activity was evaluated using surface electromyography (sEMG) with phase-specific activation metrics, and cortical activity was measured using electroencephalography (EEG) and further analyzed using spectral analysis and partial directed coherence (PDC)-based graph-theoretical measures to assess frequency-specific functional connectivity. Individuals with sarcopenia exhibited significantly reduced gait speed and shorter step length, along with prolonged loading response and pre-swing phases. Among the recorded muscles, the tibialis anterior (TA) showed significant alterations, characterized by an increased and earlier first activation peak and a reduced and delayed second peak during the gait cycle. Phase-specific analysis revealed increased TA activity during the loading response phase and decreased activity during the pre-swing phase. EEG analysis revealed beta-band-specific alterations, with increased node strength and node degree in the frontal and central regions and elevated node strength in the parietal region, while no significant differences were observed in the delta, theta, alpha, or gamma bands. These findings suggest that sarcopenia is associated with neuromuscular alterations. The coexistence of increased beta-band functional connectivity strength and persistent gait impairment may reflect inefficient compensation, in which increased neural recruitment does not fully restore gait function. These results highlight the importance of targeting neuromuscular coordination in rehabilitation.

## 1. Introduction

Sarcopenia is a progressive age-related condition characterized by the loss of skeletal muscle mass and function, and is strongly associated with impaired mobility, increased risk of falls, and reduced quality of life [1,2,3]. With the rapid aging of the global population, sarcopenia has emerged as a major clinical and public health concern [4]. Among its functional consequences, gait impairment is one of the most critical manifestations, as it directly reflects motor performance and is closely linked to adverse outcomes such as frailty, disability, and hospitalization [5,6,7]. However, despite extensive research on muscle loss, the mechanisms underlying gait dysfunction in sarcopenia remain incompletely understood, particularly from an integrated neuromuscular perspective.

Gait function involves coordinated interactions between peripheral muscle activity and central nervous system control [8,9]. During walking, motor commands originate in cortical regions, are transmitted via corticospinal pathways to lower-limb muscles, and are continuously refined by sensory feedback [10,11]. Traditionally, gait abnormalities in sarcopenia have been primarily attributed to peripheral muscular weakness [12,13]. However, increasing evidence suggests that age-related motor dysfunction may also involve altered cortical involvement rather than being solely driven by peripheral deficits. For example, Sha et al. reported that sarcopenia may be associated with an increased risk of cognitive impairment [14]. Furthermore, advancing age is associated with profound remodeling of the central nervous system, including the degradation of white matter integrity and a reduction in neurotransmitter efficiency, which inevitably disrupts the temporal precision of descending motor drives [15,16]. This indicates that the pathophysiology of sarcopenia extends beyond the boundaries of muscular atrophy, encompassing a systemic decline in the neural networks responsible for locomotion. Nevertheless, the specific neural mechanisms underlying gait dysfunction in sarcopenia remain unclear.

To address this gap, it is important to examine both peripheral muscle activation and cortical activity during gait. Movement requires dynamic and coordinated activation of specific lower-limb muscle groups to maintain postural stability, weight-bearing capacity, and forward propulsion during gait. Among these muscles, the tibialis anterior (TA), the primary ankle dorsiflexor, plays a critical role during heel strike and the swing phase by controlling foot clearance and ankle stability. In contrast, proximally located muscles such as the rectus femoris and biceps femoris are primarily responsible for hip and knee joint stabilization as well as lower-limb flexion and extension. The gastrocnemius muscle (GL) is associated with ankle propulsion during the single stance and terminal stance phase. Disruptions in the timing or magnitude of activation of these muscles may directly impair gait stability and locomotor efficiency [17]. Surface electromyography (EMG) provides insights into neuromuscular control strategies, particularly in muscles that are critical for gait stability and phase-specific control [18]. Age-related muscle loss predominantly affects the lower limbs, contributing to impaired locomotor function and increased fall risk [19,20].

Meanwhile, electroencephalography (EEG) enables the assessment of cortical activity during movement [21]. Importantly, investigating central or peripheral mechanisms in isolation fails to capture the dynamic, bidirectional “corticomuscular coupling” that governs human walking. The central nervous system constantly adapts to peripheral muscular changes by reorganizing cortical networks, a phenomenon known as neural plasticity or compensation [22,23]. Conversely, primary age-related muscle decline can destabilize the precision of these descending neural drives. Therefore, concurrent and multimodal recording of EEG and sEMG during actual ambulation is indispensable to unravel how the aging brain communicates with compromised effectors in sarcopenic states. Among different frequency bands, beta-band activity has been consistently linked to corticospinal drive and sensorimotor integration during motor tasks [24]. Alterations in beta-band activity have been reported in motor-related conditions and may reflect changes in cortical involvement during movement [25]. Therefore, integrating lower-limb muscle activation with beta-band cortical activity may provide a more comprehensive understanding of neuromuscular alterations underlying gait dysfunction in sarcopenia.

The aim of this study was to investigate gait dysfunction in sarcopenia from a neuromuscular perspective using an integrated approach combining gait analysis, surface EMG, and EEG measurements in older adults aged 65 years and older. We examined changes in gait parameters, muscle activation patterns, and frequency-specific cortical functional connectivity. To isolate the specific impact of sarcopenia on gait and neuro-motor control, potential individual confounding factors—including gender distribution, age matching, and body weight—were rigorously balanced between the cohorts, while individuals with confounding neurological or musculoskeletal comorbidities were systematically excluded. We hypothesized that sarcopenia is associated with altered peripheral muscle activation (particularly in ankle-modulating muscles) and a concurrent increase in beta-band cortical involvement, which may reflect a central compensatory response to impaired peripheral motor control. Understanding this specific pattern of neuromuscular alteration may provide new insights into the pathophysiology of sarcopenia and support the development of objective, neural-targeted strategies for rehabilitation.

## 2. Materials and Methods

### 2.1. Subjects

This study was approved by the Ethics Committee of Shanghai Sixth People’s Hospital (Approval No.: 2023-075-(1), Clinical Registration No. ChiCTR2500102516). A total of 28 participants, including 13 healthy controls (HCs) and 15 patients with sarcopenia (SAR), were recruited from the Department of Geriatrics at Shanghai Sixth People’s Hospital. Due to the exploratory nature of this preliminary pilot trial and the high technical complexity involved in continuous, synchronized ambulatory EEG-sEMG recordings in geriatric populations, a convenience sample based on clinical availability was utilized. All participants provided written informed consent prior to data collection.

Inclusion criteria were as follows:

1. Patients diagnosed with sarcopenia based on the criteria of the Asian Working Group on sarcopenia (AWGS 2019), combined with clinical manifestations;
2. Elderly people aged 65 years and above, regardless of gender;
3. Informed consent: voluntarily participated in the study and provided written informed consent prior to enrollment.

Exclusion criteria were as follows:

1. A history of neurological disorders (including stroke, Parkinson’s disease, or vestibular vertigo) or severe orthopedic conditions (such as severe knee osteoarthritis) that significantly alter normal gait, posture, or balance;
2. Presence of cognitive dysfunction or severe speech impediments that prevent the participant from understanding experimental instructions;
3. Active malignancy or a history of cancer that severely impacts physical function or muscle metabolism;
4. A history of lower-limb fracture or major lower-limb orthopedic surgery within the past 10 years.

### 2.2. Assessment of Sarcopenia

Sarcopenia was diagnosed adhering to the Asian Working Group for Sarcopenia (AWGS 2019) criteria. Appendicular skeletal muscle mass was evaluated via multi-frequency bioelectrical impedance analysis (InBody S10, InBody Co., Ltd., Seoul, Republic of Korea) and normalized by height squared to calculate the ASMI. Low muscle mass was defined as ASMI < 7.0 kg/m^2^ for men and <5.7 kg/m^2^ for women. Maximum handgrip strength was recorded using a digital dynamometer (Camry EH101, Camry Electronic Co., Ltd., Zhongshan, China) after three standing/sitting trials, with muscle weakness defined as <28 kg for men and <18 kg for women. Physical performance was assessed using a 6 m usual gait speed test (cutoff < 1.0 m/s). According to the AWGS 2019 consensus, participants were stratified based on phenotype combinations: “Sarcopenia” was defined as low muscle mass plus either low handgrip strength or slow gait speed [13].

### 2.3. Experimental Protocol

**Figure 1:**
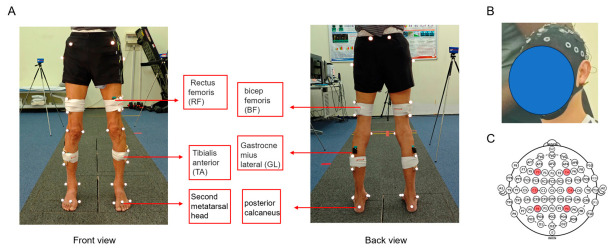
(**A**) Placement of reflective markers and surface electromyography (EMG) electrodes. (**B**) Participant wearing the electroencephalography (EEG) cap. (**C**) Schematic diagram of the EEG channel layout.

All experiments were conducted in the Sino-UK Human Performance Laboratory at Shanghai Jiao Tong University. Participants were instructed to walk along a 10 m pathway at their self-selected comfortable speed. Although a total of 20 walking trials were initially collected for each participant, only 8 trials with the most consistent walking velocities were retained for subsequent analysis to ensure data stationarity. Specifically, trials were ranked by average walking speed, and the 8 trials with the smallest deviation from the median speed were selected. For each subject, four infrared-reflexive markers were placed on the heel and the second toe positions of the feet, which were used to get the gait events including heel strike and toe off. The trajectories of these four markers were collected by an optical motion capture system (Vicon Nexus 1.8.5, Oxford Metrics, Oxford, UK) with eight cameras in a sampling frequency of 100 Hz. Eight surface electromyography (sEMG) sensors (Trigno Wireless System, Delsys Inc., Boston, MA, USA) were placed over the muscle bellies of the tibialis anterior (TA), gastrocnemius lateralis (GL), rectus femoris (RF), and biceps femoris (BF) bilaterally. The sEMG signals were acquired using the Trigno Control Utility software (version 3.6.0, Delsys Inc., MA, USA) at a sampling rate of 2000 Hz. All sensors were placed based on the SENIAM guidelines [26]. EEG signals were recorded using a 64-channel wireless system (NSW64, Neuracle Technology Co., Ltd., Changzhou, China) at 1000 Hz. Conductive gel was applied to ensure electrode impedance <10 kΩ (Figure 1). The EEG, EMG, and 3D motion capture systems were synchronized via an event trigger: a timing pulse sent at trial onset enabled precise temporal alignment of all signals during post-processing.

### 2.4. Data Processing

EMG and EEG were processed using Python 3.13. Gait events including heel strike (HS) and toe off (TO) were calculated based on the trajectory data of the infrared reflective markers of the heel and the second toe [27]. Gait cycle was divided into four phases: loading response phase, single stance phase, pre-swing phase and swing phase. Spatiotemporal parameters including cadence (steps/min), step length (m), step time (s), gait phase time (s), walking speed (m/s) and step width (m) were calculated.

Since sEMG signals were collected bilaterally from homologous muscle pairs (TA, GL, RF, and BF), the averaged values of the left and right limbs were calculated and used for all subsequent analyses. This approach was adopted to represent symmetric lower-limb muscle activation patterns during steady-state walking and to reduce potential side-related variability. Raw sEMG signals were visually inspected to exclude trials contaminated by artifacts or signal corruption. Valid signals then underwent detrending and DC removal, followed by band-pass filtering using a 4th-order zero-lag Butterworth filter (20–500 Hz). sEMG envelopes were extracted using a moving RMS procedure with a 100 ms sliding window. Specifically, signals were squared and smoothed using a moving-average window corresponding to 100 ms. To reduce inter-subject variability and facilitate comparison of temporal activation profiles, sEMG amplitudes were normalized within each gait cycle by dividing each time point of the sEMG envelope by the mean envelope amplitude of the corresponding gait cycle (within-cycle mean normalization). After normalization, each gait cycle was resampled to 100 points representing 0–100% of the gait cycle. Consequently, normalized sEMG values were dimensionless and expressed in arbitrary units (a.u.).

EEG preprocessing and artifact rejection: Raw EEG data were preprocessed using MNE-Python. The pipeline included: (1) automatic bad channel detection followed by spherical interpolation; (2) band-pass filtering (0.5–50 Hz) combined with a 50 Hz notch filter to suppress line noise; and (3) Independent Component Analysis (FastICA algorithm) for source decomposition. ICA components were manually screened based on standardized neurophysiological criteria. Ocular artifacts were identified by frontal-polar scalp topographies and time courses synchronized with blink or saccade activity. Gait-related motion and muscle artifacts were identified by widespread or focal topographies, particularly over temporal and occipital regions, elevated high-frequency spectral content (>30 Hz), and transient large-amplitude deflections phase-locked to heel strikes. Components meeting these criteria were excluded prior to signal reconstruction. On average, approximately 4–6 ICA components per participant were removed, with the majority corresponding to ocular and gait-related motion artifacts. Following ICA reconstruction, EEG data were segmented into epochs time-locked to consecutive left heel strikes (LHS), representing individual gait cycles. Finally, sensorimotor cortical activity was extracted from electrodes F3, F4, C3, C4, P3, and P4 for subsequent analysis.

To investigate cortical dynamics during gait in older adults with sarcopenia, electroencephalographic (EEG) signals were analyzed using a multi-modal approach encompassing spectral and functional connectivity features. Power spectral density (PSD) was estimated using Welch’s method, an improved periodogram approach that reduces spectral variance through signal segmentation, windowing, and averaging. At the same time, differences in skull thickness, electrode impedance, and brain structure among individuals could lead to significant variations in absolute PSD levels; therefore, we used the widely accepted Theta–Alpha Ratio (TAR) as a sensitive biomarker reflecting cognitive function [28,29,30].

Partial directed coherence (PDC) was used to assess directional information flow between brain regions in the frequency domain during walking. PDC is based on a multivariate autoregressive (MVAR) model of order p:

$$x\left(t\right)=\sum_{k=1}^{p}A\left(k\right)x\left(t-k\right)+e\left(t\right)$$

where $x\left(t\right)\in R^{m}$ is the EEG signal vector at time t, $A\left(k\right)$ is the m×m coefficient matrix at lag k, and $e\left(t\right)$ is the residual noise. The frequency-domain representation is:

$$A\left(f\right)=I-\sum_{k=1}^{p}A(k)e^{-i2\pi fk/f_{s}}$$

PDC from channel j to channel i at frequency f  is defined as:

$$\pi_{ij}\left(f\right)=\frac{{|A}_{ij}\left(f\right)|}{\sqrt{\sum_{k=1}^{m}{{|A}_{kj}\left(f\right)|}^{2}}}$$

where $A_{ij}\left(f\right)$ is the (i,j)−th element of  A(f), and the denominator normalizes the influence of source j across all targets. PDC values range from 0 to 1, reflecting the relative strength of directed connectivity.

To eliminate spurious connections arising from volume conduction or random fluctuations, a significance test was performed on the PDC network. Phase-randomized surrogate data were generated by applying a fast Fourier transform (FFT) to each original time series, preserving the amplitude spectrum while replacing the phase with uniformly distributed random values, followed by an inverse FFT to reconstruct the surrogate signals. Given that PDC quantifies directed coupling based on inter-signal phase relationships, genuine effective connectivity should significantly exceed the connection strengths estimated from the surrogate data. Consequently, only PDC values that passed the significance threshold were retained for subsequent graph-theoretical analysis, with non-significant connections set to zero. The thresholded network was subsequently analyzed as a weighted network.

Based on the PDC-weighted functional networks, four graph-theoretical metrics were extracted to evaluate brain network organization at local levels:

(1) Node Strength: the sum of connection weights (PDC values) linked to a node, indicating its overall involvement in information exchange.
(2) Node Degree: the number of significant connections to a node, reflecting its topological centrality.
(3) Clustering Coefficient: measures the extent to which a node’s neighbors are interconnected, reflecting local functional segregation.
(4) Local Efficiency: the efficiency of information transfer within a node’s neighborhood, indicating network resilience.

### 2.5. Statistical Analysis

Statistical analyses were performed using SPSS software (version 25.0; IBM Corp., Chicago, IL, USA) and GraphPad Prism software (version 10.4.1; GraphPad Software, San Diego, CA, USA). Continuous variables—including demographic profiles, spatiotemporal gait parameters, sEMG root-mean-square (RMS) envelopes, and EEG frequency values—were first evaluated for normality using the Shapiro–Wilk test. Formally, normally distributed data were expressed as Mean ± SD and analyzed using an independent samples Student’s *t*-test to evaluate differences between the Sarcopenia (SAR) and Healthy Control (HC) groups. Non-normally distributed parameters were evaluated utilizing the non-parametric Mann–Whitney U test. Categorical clinical characteristics, including gender distribution and comorbidity prevalence (hypertension, non-lower limb osteoarthritis, and medication profiles), were presented as frequencies and percentages [*n* (%)] and compared using the Chi-square test or Fisher’s exact test. For all statistical verifications, the significance threshold was strictly set at *p* < 0.05.

## 3. Results

### 3.1. Baseline Characteristics of Participants

At baseline, no significant between-group differences were observed regarding gender (*p* = 0.937), age (*p* = 0.102), or height (*p* = 0.377). However, compared to healthy controls, individuals with sarcopenia exhibited significantly lower body weight (*p* = 0.013), reduced ASMI (*p* < 0.005), and impaired handgrip strength (*p* < 0.005). Regarding clinical baselines, the primary comorbidities across the cohort were hypertension and non-lower limb osteoarthritis, with antihypertensive agents and non-steroidal anti-inflammatory drugs (NSAIDs) being the most prevalent medications; notably, the distribution of both comorbidities and concomitant medications was statistically comparable between the two groups (all *p* > 0.05) (Table 1).

### 3.2. Gait Spatiotemporal Parameters

The analysis of gait spatiotemporal parameters showed that the step length and walking speed of patients with sarcopenia were significantly lower than those of the healthy control group (*p* = 0.01, *p* < 0.005). Compared with the healthy control group, the loading response period and pre-swing period of patients with sarcopenia were significantly prolonged (*p* = 0.043, *p* = 0.036) (Table 2).

### 3.3. Muscle Activity Results

Surface EMG analysis revealed that among the four recorded muscles (gastrocnemius lateralis, rectus femoris, biceps femoris, and tibialis anterior (TA)), only the TA exhibited significant changes, while no consistent differences were observed in the other muscles between groups.

**Figure 2:**
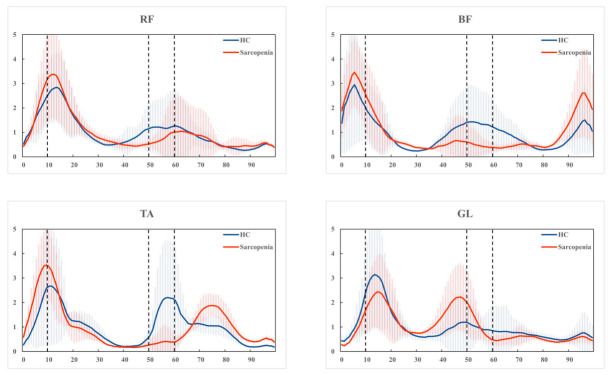
The sEMG profiles of the four muscles—specifically the gastrocnemius lateralis (GL), rectus femoris (RF), biceps femoris (BF), and tibialis anterior (TA)—are plotted as a percentage of the normalized gait cycle (0% to 100%). sEMG amplitudes were normalized to the mean envelope amplitude of each gait cycle; therefore, *y*-axis values represent normalized RMS EMG amplitudes in arbitrary units (a.u.). The blue solid lines and shaded areas represent the Mean ± SD of the Healthy Control group (HC, n = 13), while the red solid lines and shaded areas represent the Sarcopenia group (SAR, n = 15). Vertical dashed lines delineate the four core gait phases: Loading Response (LR), Single Stance (SS), Pre-Swing (PS), and Swing Phase (SW).

The TA muscle demonstrated a characteristic biphasic activation pattern during the gait cycle. In the sarcopenia group, the first activation peak was significantly increased (*p* = 0.009) and occurred earlier compared to the control group (*p* < 0.001). In contrast, the second peak was reduced (*p* = 0.004) and showed a delayed timing (*p* < 0.016). These findings indicate a disruption in the temporal coordination of TA activation (Figure 2) (Table 3).

Further analysis across gait phases revealed phase-specific differences in TA activity. The root mean square (RMS) value of TA was significantly increased during the loading response (LR) phase (*p* = 0.001), whereas it was significantly decreased during the pre-swing (PS) phase in the sarcopenia group (*p* = 0.001). No significant differences were observed in the single stance (SS) and swing (SW) phases (Table 4).

### 3.4. EEG Analysis

No significant differences were observed in node strength, node degree, clustering coefficient, or local efficiency across delta, theta, and alpha bands in the frontal, central, and parietal regions between the sarcopenia and control groups. Further analysis revealed no significant differences were observed in the theta/alpha ratio (TAR) across frontal, central, and parietal regions between the sarcopenia and control groups. These findings suggest that global cortical activity and general cognitive state were comparable between groups (Table 5).

**Figure 3:**
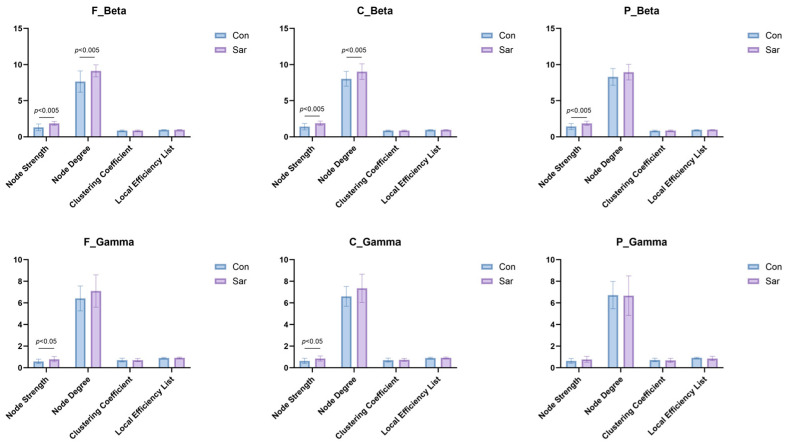
Comparison of EEG power spectral parameters in the beta and gamma bands across distinct cortical regions during gait. The bar plots illustrate the localized neurophysiological alterations between the Healthy Control group (HC, n = 13, represented by blue bars) and the Sarcopenia group (SAR, n = 15, represented by purple bars).

In the beta band (13–30 Hz), significant increases in node strength and node degree were observed in the sarcopenia group compared with controls. Specifically, both node strength and node degree were significantly elevated in the frontal and central regions, while the parietal region showed a significant increase in node strength. No significant differences were found in clustering coefficient or local efficiency between groups, indicating that network alterations were primarily reflected in connectivity strength rather than global network topology. In the gamma band (30–50 Hz), a significant increase in node strength was observed in the frontal and central regions, while other network metrics remained unchanged (Figure 3).

### 3.5. Correlation Between Cortical Connectivity and Gait Impairment

**Figure 4:**
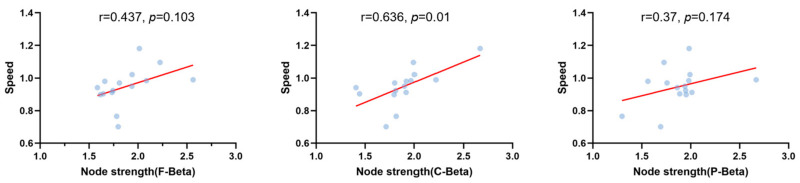
Intra-group correlation trajectories between beta-band cortical node strength and walking speed within the sarcopenia group.

To elucidate the clinical correlation between neurophysiological alterations and motor decline, a within-group correlation analysis was conducted exclusively within the sarcopenia group. The results revealed that the beta-band node strength in the central region was significantly positively correlated with patients’ walking speed (r = 0.636, *p* = 0.01). From an individual perspective, patients with higher node strength exhibited superior gait preservation. In contrast, the correlations within the frontal and parietal cortical areas fell short of statistical significance, yet consistently maintained parallel positive trajectories (Figure 4).

## 4. Discussion

In this study, we assessed gait characteristics, muscle activity, and cortical functional connectivity in individuals with sarcopenia compared to healthy controls using motion capture, surface EMG, and EEG analysis. Compared with controls, individuals with sarcopenia showed reduced walking speed and shorter step length, consistent with previous studies [13,31,32,33]. Surface EMG analysis revealed that only the tibialis anterior exhibited significant alterations, characterized by disrupted activation timing and phase-dependent changes in RMS. In addition, EEG analysis demonstrated increased beta-band functional connectivity strength in frontal, central, and parietal regions, while network topology remained unchanged. Taken together, these findings suggest that sarcopenia is associated with concurrent alterations in gait performance, muscle activation patterns, and beta-band functional connectivity strength.

A notable finding of this study is that gait impairment in sarcopenia was not limited to global spatiotemporal decline, but also involved phase-specific alterations. The prolongation of loading response and pre-swing suggests that gait dysfunction in sarcopenia may reflect impaired weight acceptance and reduced transition efficiency from stance to swing [34,35]. Many gait disorders are characterized by an extended double-support phase in the early stage, which is aimed at enhancing walking stability, patterns commonly interpreted as a more cautious and less efficient walking strategy [36,37]. In this context, prolonged early and late stance subphases may indicate that individuals with sarcopenia require more time to stabilize the body after initial contact and to generate adequate propulsion before limb advancement.

At the peripheral level, the most distinctive EMG finding was the selective alteration of tibialis anterior activity, whereas the other recorded muscles (RF, BF, GL) did not show consistent between-group differences. This muscle-specific pattern is important, because the tibialis anterior plays a central role in controlling ankle dorsiflexion during limb advancement and in modulating foot positioning at initial contact [38,39,40]. In the present study, the earlier and enhanced first peak, together with the delayed and attenuated second peak, indicates a disruption in the normal temporal coordination of tibialis anterior recruitment. The phase-specific RMS pattern further supports this interpretation: increased activity during loading response may reflect a greater demand for stabilization during early stance, whereas reduced activity during pre-swing may indicate impaired preparation for limb transition. Together, these findings suggest that sarcopenia-related gait impairment may be linked to altered phase-dependent activation of the tibialis anterior, which may in turn affect ankle control during walking [41,42,43], rather than reflecting a generalized alteration of all lower-limb muscles.

The cortical findings provide an additional and clinically meaningful layer of interpretation. No significant differences were observed in delta, theta, alpha, or TAR measures, suggesting that neural alterations in sarcopenia are unlikely to reflect global cortical changes [44,45]. Instead, the most robust differences emerged in the beta band, where node strength and node degree increased mainly in frontal and central regions, with additional increases in parietal strength. This result aligns with previous work showing that beta oscillatory activity has been repeatedly linked to corticospinal contributions to walking and motor output [46,47]. In contrast, only limited and isolated differences were observed in the gamma band, suggesting that high-frequency activity may not represent a dominant neural feature in this context. Furthermore, the absence of group differences in clustering coefficient and local efficiency indicates that the overall network topology remained relatively preserved. Thus, the principal neural change in sarcopenia in this dataset was not a wholesale reorganization of network architecture, but rather an increase in the strength and extent of functional interactions within a largely preserved network scaffold.

Importantly, these peripheral and cortical findings should not be interpreted in isolation. To bridge the gap between supraspinal alterations and behavioral deficits, we performed a direct correlation analysis, which unraveled that beta-band network node strength in the central region was significantly and positively coupled with usual walking velocity within the sarcopenia cohort (r = 0.636, *p* < 0.05). This quantitative brain–behavior relationship provides direct evidence that altered cortical connectivity is intertwined with actual ambulatory performance. However, given the cross-sectional, observational design of this study, caution is mandatory against drawing definitive causal inferences. The coexistence of phase-specific gait slowing and increased beta-band connectivity presents a clear statistical correlation, which may be interpreted as a framework of inefficient compensation. Increased cortical involvement may represent an adaptive neural correlate to offset declining peripheral motor control; however, this increased neural recruitment did not normalize gait performance. Importantly, we cannot rule out the alternative hypothesis that this hyper-connectivity fundamentally reflects age-related motor network degradation or a pathological, inefficient scattering of synchronized neural resources, rather than a purposeful compensatory effort. Ultimately, our observational data capture a robust neural engagement concomitant with gait dysfunction, but tracking whether this central shift represents a top-down survival strategy or a symptom of systemic neuromuscular decay requires longitudinal or interventional validation. Previous studies have shown that older adults frequently rely more on supraspinal resources during walking and balance, particularly when automatic motor control becomes less efficient [48,49].

From a translational perspective, the cortical–behavioral coupling unraveled in this exploratory study offers preliminary insights for future neurorehabilitation design. Our findings imply that future clinical interventions might benefit from transitioning from conventional peripheral training toward central–peripheral modulation strategies. For therapists, exploring innovative motor–sensory integration protocols could be a promising avenue to optimize neural resource mobilization and mitigate potential central overloads. For patients, structured coordination exercises might serve as a potential approach to facilitate active top-down cortical compensation. Nevertheless, these conceptual frameworks represent early-stage theoretical paths that require systematic verification in future large-scale trials.

Several limitations should be acknowledged in this study. First, the sample size was relatively small, and the cross-sectional design precludes causal inference. Crucially, due to the exploratory nature and restricted sample size of this pilot trial, a statistically powered gender-stratified sub-analysis could not be performed, although gender was stringently matched at baseline to control for potential confounding effects. Second, only a limited number of muscles and network metrics were analyzed, which may not fully capture the complete profile of neuromuscular coordination. Future studies should incorporate larger, sex-balanced cohorts and explore multi-frequency brain–muscle relationship metrics to further clarify the role of beta-band connectivity in functional outcomes and refine targeted neurorehabilitation protocols.

## 5. Conclusions

This study suggests that sarcopenia-related gait dysfunction is associated with peripheral motor impairment alongside increased beta-band cortical involvement. While functional connectivity strength was elevated, network topology remained preserved, indicating increased neural engagement without structural reorganization. Importantly, gait performance remains impaired despite enhanced cortical involvement, supporting the presence of inefficient compensation. These findings highlight a mismatch between neural effort and motor function and suggest that improving neuromuscular efficiency may be critical for intervention. Nevertheless, given the limited sample size of this exploratory cohort, these preliminary insights must be interpreted with caution, as they cannot be widely generalized prior to large-scale clinical validation.

## Figures and Tables

**Table 1:** Baseline characteristics of participants.

Category	Group		*p*
	HC (*n* = 13)	SAR (*n* = 15)	
Gender (Male/Female)	5/8	6/9	0.937
Age	72.21 ± 4.95	75.85 ± 6.14	0.102
Height (cm)	162.96 ± 9.30	159.81 ± 8.93	0.377
Weight (kg)	63.44 ± 12.63	52.26 ± 8.43	0.013
ASMI	6.87 ± 0.78	5.68 ± 0.60	<0.005
Grip strength	28.28 ± 3.26	21.97 ± 4.22	<0.005
Comorbidities			
Hypertension	4 (30.7%)	6 (40%)	0.705
Non-lower limb osteoarthritis	2 (15.3%)	4 (26.6%)	0.651
Medications			
Antihypertensive drugs	4 (30.7%)	6 (40%)	0.705
NSAIDs	1 (7.6%)	3 (20%)	0.601

Note: Data are presented as Mean ± SD (standard deviation) for continuous variables or as number (percentage) for categorical variables. HC, healthy control; SAR, sarcopenia; ASMI, appendicular skeletal muscle mass index; NSAIDs, non-steroidal anti-inflammatory drugs.

**Table 2:** Gait spatiotemporal parameters.

Parameters	HC	SAR	*p* Value
Cadence (steps/min)	116.31 ± 9.21	106.88 ± 8.49	0.070
Step length (m)	0.61 ± 0.04	0.53 ± 0.06	0.010
Step time (s)	0.52 ± 0.04	0.57 ± 0.04	0.062
Gait phase time (s)			
LR	0.09 ± 0.02	0.11 ± 0.02	0.043
SS	0.43 ± 0.02	0.45 ± 0.03	0.135
PS	0.09 ± 0.02	0.12 ± 0.02	0.036
SW	0.43 ± 0.02	0.45 ± 0.03	0.176
Walking speed (m/s)	1.19 ± 0.14	0.95 ± 0.12	<0.005
Step width (m)	0.11 ± 0.02	0.09 ± 0.02	0.141

Note: Data are presented as Mean ± SD (standard deviation) for continuous variables. HC, healthy control; SAR, sarcopenia; LR, Loading Response; SS, Single Stance; PS, Pre-Swing; SW, Swing Phase.

**Table 3:** sEMG Peak parameters in SAR patients and HC.

	Group		*p*
	HC (*n* = 13)	SAR (*n* = 15)	
RF			
PA_1	4.32 ± 1.50	4.31 ± 1.79	0.988
PL_1	22.61 ± 12.08	18.35 ± 12.48	0.376
PA_2	2.61 ± 1.80	1.75 ± 1.48	0.195
PL_2	18.62 ± 7.72	22.24 ± 8.79	0.268
BF			
PA	5.63 ± 1.29	6.02 ± 0.90	0.374
PL	33.89 ± 20.09	34.29 ± 21.59	0.961
TA			
PA_1	2.96 ± 1.63	4.56 ± 1.07	0.009
PL_1	20.54 ± 5.69	10.53 ± 4.65	<0.001
PA_2	4.54 ± 1.66	2.85 ± 0.62	0.004
PL_2	14.47 ± 7.64	21.70 ± 6.84	0.016
GL			
PA	5.31 ± 1.58	5.20 ± 1.04	0.848
PL	36.10 ± 18.47	35.75 ± 13.33	0.955

Note: Data are presented as Mean ± SD (standard deviation) for continuous variables. HC, healthy control; SAR, sarcopenia; RF, Rectus Femoris; BF, Biceps Femori; TA, Tibialis Anterior; GL, Gastrocnemius Lateralis.

**Table 4:** sEMG parameters in SAR patients and HC.

	Group		*p*
	HC (*n* = 13)	SAR (*n* = 15)	
RF			
LR	1.72 ± 0.97	2.04 ± 1.13	0.443
SS	1.77 ± 0.63	1.79 ± 0.67	0.944
PS	1.34 ± 1.22	0.78 ± 0.96	0.204
SW	0.88 ± 0.55	0.76 ± 0.58	0.597
BF			
LR	2.41 ± 2.03	2.94 ± 1.12	0.416
SS	1.33 ± 0.54	1.35 ± 0.54	0.917
PS	1.44 ± 1.47	0.51 ± 0.66	0.053
SW	1.19 ± 0.39	1.41 ± 0.61	0.279
TA			
LR	0.92 ± 1.14	2.55 ± 0.98	0.001
SS	1.25 ± 0.58	1.42 ± 0.53	0.440
PS	2.41 ± 1.68	0.38 ± 0.69	0.001
SW	1.47 ± 0.63	1.29 ± 0.36	0.412
GL			
LR	1.34 ± 1.00	0.93 ± 0.65	0.226
PS	1.18 ± 0.89	1.32 ± 0.79	0.663
SS	1.94 ± 0.95	2.15 ± 0.56	0.500
SW	0.86 ± 0.61	0.63 ± 0.42	0.290

Note: Data are presented as Mean ± SD (standard deviation) for continuous variables. HC, healthy control; SAR, sarcopenia; LR, Loading Response; SS, Single Stance; PS, Pre-Swing; SW, Swing Phase; RF, Rectus Femoris; BF, Biceps Femori; TA, Tibialis Anterior; GL, Gastrocnemius Lateralis.

**Table 5:** EEG parameters in SAR patients and HC.

	Group		*p*
	HC (*n* = 13)	SAR (*n* = 15)	
F_ Delta (0.5–4 Hz)			
Node Strength	1.23 ± 0.34	1.53 ± 0.42	0.053
Node Degree	6.50 ± 1.22	6.93 ± 1.34	0.396
Clustering Coefficient	0.79 ± 0.10	0.76 ± 0.08	0.441
Local Efficiency	0.94 ± 0.05	0.95 ± 0.04	0.777
C_Delta (0.5–4 Hz)			
Node Strength	1.36 ± 0.49	1.68 ± 0.51	0.109
Node Degree	6.85 ± 0.99	7.46 ± 1.34	0.187
Clustering Coefficient	0.78 ± 0.13	0.75 ± 0.08	0.455
Local Efficiency	0.93 ± 0.05	0.94 ± 0.05	0.810
P_Delta (0.5–4 Hz)			
Node Strength	1.31 ± 0.47	1.57 ± 0.47	0.153
Node Degree	6.65 ± 0.92	6.96 ± 1.23	0.468
Clustering Coefficient	0.79 ± 0.09	0.79 ± 0.08	0.983
Local Efficiency	0.94 ± 0.04	0.96 ± 0.04	0.164
F_Theta (4–8 Hz)			
Node Strength	1.20 ± 0.32	1.43 ± 0.28	0.057
Node Degree	6.69 ± 0.88	7.54 ± 1.25	0.053
Clustering Coefficient	0.80 ± 0.09	0.77 ± 0.07	0.364
Local Efficiency	0.94 ± 0.04	0.96 ± 0.04	0.276
C_Theta (4–8 Hz)			
Node Strength	1.23 ± 0.42	1.48 ± 0.35	0.098
Node Degree	6.62 ± 1.23	7.54 ± 1.42	0.085
Clustering Coefficient	0.80 ± 0.10	0.78 ± 0.06	0.507
Local Efficiency	0.94 ± 0.05	0.96 ± 0.03	0.272
P_Theta (4–8 Hz)			
Node Strength	1.26 ± 0.45	1.43 ± 0.32	0.269
Node Degree	6.85 ± 1.01	7.21 ± 1.03	0.358
Clustering Coefficient	0.81 ± 0.09	0.80 ± 0.07	0.792
Local Efficiency	0.95 ± 0.04	0.97 ± 0.03	0.127
F_Alpha (8–13 Hz)			
Node Strength	1.25 ± 0.37	1.43 ± 0.32	0.197
Node Degree	7.31 ± 1.44	7.46 ± 1.32	0.770
Clustering Coefficient	0.75 ± 0.22	0.81 ± 0.07	0.318
Local Efficiency	0.90 ± 0.19	0.97 ± 0.03	0.177
C_Alpha (8–13 Hz)			
Node Strength	1.27 ± 0.46	1.53 ± 0.32	0.101
Node Degree	7.19 ± 1.52	7.79 ± 1.10	0.255
Clustering Coefficient	0.80 ± 0.12	0.82 ± 0.07	0.533
Local Efficiency	0.95 ± 0.05	0.97 ± 0.03	0.248
P_Alpha (8–13 Hz)			
Node Strength	1.28 ± 0.36	1.46 ± 0.24	0.134
Node Degree	7.27 ± 1.22	7.46 ± 0.75	0.617
Clustering Coefficient	0.80 ± 0.12	0.82 ± 0.06	0.475
Local Efficiency	0.95 ± 0.05	0.97 ± 0.03	0.210
TAR			
F	0.17 ± 0.18	0.19 ± 0.16	0.709
C	0.13 ± 0.13	0.13 ± 0.15	0.946
P	0.19 ± 0.23	0.15 ± 0.14	0.611

Note: Data are presented as Mean ± SD (standard deviation) for continuous variables. HC, healthy control; SAR, sarcopenia.

## Data Availability

The datasets used and/or analyzed during the current study are available from the corresponding author on request.
